# Very High Dose Epinephrine for the Treatment of Vasoplegic Syndrome during Liver Transplantation

**Published:** 2013-02-01

**Authors:** M. B. Khosravi, S. Milani, S. Ghaffaripour, A. Sahmeddini, M. H. Eghbal, S. A. Malek-Hosseini

**Affiliations:** 1 *Shiraz Anesthesiology and Critical Care Research Center, Shiraz University of Medical Sciences, Shiraz, Iran*; 2 *Department of Organ Transplantation, School of Medicine, Shiraz University of Medical Sciences, Shiraz, Iran.*

**Keywords:** Epinephrine, Hepatocellular carcinoma, Liver transplantation, Vasoplegia

## Abstract

A 55-year-old man with hepatitis B and hepatocellular carcinoma was treated with liver transplantation without veno-venous bypass. During the procedure his arterial blood pressure remained at 55/30 mm Hg and did not respond to increasing doses of norepinephrine. Vasoplegia was managed aggressively with the intravenous infusion of high doses of epinephrine.

## INTRODUCTION

Norepinephrine-refractory vasoplegia (vasoplegic syndrome) has been reported in cardiac surgery [1]; the condition is similar to septic shock [2]. In the setting of liver transplantation, vasoplegic syndrome shares the hemodynamic characteristics of low systemic vascular resistance and high cardiac output. The mechanisms of refractory vasoplegia involve profound vasodilatation induced by nitric oxide and cyclic guanosine monophosphate [3]. However; vasoplegic syndrome differs from post-reperfusion syndrome in terms of its symptoms, etiology, and treatment [4].

## CASE REPORT

A 55-year-old man (weight 80 kg, height 178 cm) with hepatitis B and hepatocellular carcinoma was treated with liver transplantation without veno-venous bypass. He was ASA status III and MELD score 25. His preoperative serum creatinine was 1.08 mg/dL. Routine monitoring was established, then anesthesia was induced. Anesthesia was maintained with isoflurane, morphine and atracurium (intravenous bolus doses).

Approximately, 30 min after initiation of the operation, his arterial blood pressure rapidly dropped to 50/30 mm Hg. Infusion of norepinephrine (0.1–0.8 µg/kg/min) and dopamine (2.0 µg/kg/min) was started. After 45 min, the patient had still poor response to increasing doses of norepinephrine; the cardiac output was high (8.0 L/min), and the systemic vascular resistance was low (460 dyn s/cm^5^). Therefore, epinephrine was administered at a rate of 0.9 µg/kg/min; dopamine was also given at a rate of 2.0 µg/kg/min. Fluids were administered via rapid infusion system to maintain a central venous pressure in the range of 8–12 mm Hg.

The anhepatic phase took 68 min. After the suprahepatic and infrahepatic vena cava and portal vein were unclaimed, the patient's need for epinephrine decreased.

Urine output was less than 1 mL/kg/min although the central venous pressure was maintained above 8 mm Hg and dopamine, furosemide and N-acetylcysteine were administered during the operation. Oxygen saturation and temperature remained within the normal range. Arterial blood gases and plasma potassium, sodium and hemoglobin also remained within acceptable ranges. The total operative time was 390 min (Table 1).

**Table 1:** Evolution of the patient's intraoperative fluid intakes and outputs

**Fluids**	**Time (min)**							
	**60**	**120**	**180**	**240**	**300**	**360**	**390**	**Total**
Blood loss (mL)	ND	2000	1000	3000	200	300	ND	6500
Packed RBC (U)	ND	ND	4	4	2	3	ND	13
Fresh frozen plasma (mL)	ND	ND	1000	400	200	400	ND	2000
Albumin (g)	10	10	10	10	10	10	ND	60
Fluids (mL)*	1500	2100	2400	1000	1000	1000	1000	10000
Urinary output (mL)	ND	5	3	32	80	76	54	250

ND: No data

* 3000 mL half saline, 6000 mL normal saline

After surgery, the patient was transferred to the ICU, and epinephrine was infused in combination with dopamine 2.0 µg/kg/min to maintain the arterial blood pressure during the first 6 h of surgery. Continuous renal replacement therapy was initiated immediately post-operative in the ICU; the patient's endotracheal tube was removed 24 h later. On the first post-operative day, the serum creatinine level was 2.1 mg/dL; on the second day, it was 2.2 mg/dL. The patient was discharged from the ICU on day six with a serum creatinine level of 1.5 mg/dL. He was discharged from the hospital on post-operative day 20 without any further complications. After nine months of follow-up, the BUN and creatinine were 16 mg/dL and 1.1 mg/dL, respectively.

## DISCUSSION

Vasoplegic syndrome is generally characterized by persistent low arterial blood pressure (systolic pressure <60 mm Hg), increased cardiac output (cardiac index >2.5 L/min/m2) and decreased systemic vascular resistance (<800 dyne s/cm5). These alterations may persist despite intravenous infusion of ≥0.5 µg/kg/min norepinephrine. Vasoplegic differs from post-reperfusion syndrome in terms of its symptoms, etiology, and treatment [4-6].

The mechanism of vasoplegic syndrome is still unclear, but it may be related to surgical trauma, transfusion of blood components, liver and gastrointestinal tract ischemia, reperfusion injury, neuroendocrine disorder, systemic inflammatory response, endotoxemia, or other factors [4, 7]. The duration of vasoplegic syndrome significantly influences the outcome, so its prompt, accurate diagnosis and aggressive management are paramount to reduce its post-operative morbidity and mortality [1, 8]. Once the vasoplegia is identified, norepinphrine should be used as early as possible at a dosage up to 5.0 µg/kg/min [9]. In general, adrenalin is used when high-dose noradrenalin has failed [10]. Arginine vasopressin may be used as a supplement to catecholamines to reduce the dosage of norepinephrine or as a first-line treatment for vasoplegic syndrome [11]. In patients who do not respond to noradrenaline, methylene blue is a reasonable therapeutic choice [8, 12].

Our patient’s arterial blood pressure decreased rapidly almost 30 min after the start of the operation to 50/30 mm Hg; the pressure responded poorly to volume infusions and increasing doses of norepinephrine (>0.5 µg/kg/min). Therefore, intravenous infusion of epinephrine was started at a rate of 0.9 µg/kg/min to maintain the arterial blood pressure during the next 3 h.

Previous studies recommended methylene blue to treat vasoplagic syndrome, but this drug was not available at the time our patient underwent liver transplantation, so we opted to use high-dose epinephrine. Norepinephrine, epinephrine and endothelin-1 are important elements in renal hypoperfusion and sodium-water retention in advanced cirrhosis [13, 16]. In decompensated patients, normalization of increased arterial blood pressure may increase renal perfusion and improve renal function [13, 17]. Maintaining blood pressure during liver transplantation appears to have a positive effect on peri-operative renal function. The use of high doses of epinephrine and norepinephrine for treating vasoplegic syndrome would probably increase blood pressure and improve renal function without increasing vasoconstriction in the splanchnic and renal vasculature, and thus without compromising liver and renal function.

After successful intra-operative management of vasoplegic syndrome in the course of liver transplant surgery, our patient was discharged from the hospital after 20 days without any complications.
